# Selenite Uptake and Transformation in Rice Seedlings (*Oryza sativa* L.): Response to Phosphorus Nutrient Status

**DOI:** 10.3389/fpls.2020.00874

**Published:** 2020-06-23

**Authors:** Yaqi Wang, Kang Wang, Qi Wang, Yanan Wan, Zhong Zhuang, Yao Yu, Huafen Li

**Affiliations:** Beijing Key Laboratory of Farmland Soil Pollution Prevention and Remediation, Key Laboratory of Plant-Soil Interactions of the Ministry of Education, College of Resources and Environmental Sciences, China Agricultural University, Beijing, China

**Keywords:** selenite, phosphorus, rice, translocation, subcellular distribution, Se speciation

## Abstract

Selenite and phosphate share similar uptake mechanisms, as a phosphate transporter is involved in the selenite uptake process. However, the mechanism by which selenium (Se) transformation in plants is mediated by phosphorus (P) remains unclear. In this hydroponic study, the absorption, translocation, and biotransformation of Se in selenite-treated rice (*Oryza sativa* L.) seedlings were investigated under varying P nutrient status. The results showed that P-deficient cultivation increased the Se concentration in roots with Se-only treatment by 2.1 times relative to that of the P-normal condition. However, co-treating roots with additional P caused the Se concentration to decline by 20 and 73% compared to Se treatment alone under P-normal and P-deficient cultivation, respectively. A similar pattern was also observed in Se uptake by rice roots. With an Se-transfer factor elevated by 4.4 times, the shoot Se concentration was increased by 44% with additional P supply compared to the concentration under Se-only treatment of P deficiency; however, no significant differences were observed regarding P-normal cultivation. P deficiency increased the Se percentage by 28% within the cell wall, but reduced it by 60% in the soluble fraction of Se-only treated roots relative to that of the P-normal condition. Contrarily, compared with the Se-only treatment under P deficiency, additional P supply enhanced Se storage in the root soluble fraction by 1.3 times. The opposite tendency was observed for rice shoots. Moreover, P deficiency reduced the proportion of SeMet by 22%, but increased MeSeCys by 1.3 times in Se-only treated roots compared to those under the P-normal condition. Interestingly, MeSeCys was not detected when additional P was added to the two cultivation conditions. Unlike in the roots, only SeMet was generally detected in the rice shoots. The results demonstrate that the P nutrient status strongly affects the Se biofortification efficiency in rice seedlings by altering the Se subcellular distribution and speciation.

## Introduction

Selenium (Se), considered an essential trace element for humans and animals, is critical for antioxidation, immune system support, and disease prevention (Rayman, 2000). Se is primarily acquired by humans via their diet (Broadley et al., 2006), and plant-derived Se has thus attracted extensive attention because of its safety and bioavailability. Regrettably, the Se content of plants is generally not sufficient to meet human nutritional demands. It is estimated that approximately one billion people have suffered from Se deficiency worldwide (Stoffaneller and Morse, 2015), with Se intakes lower than the recommended value of 55–200 μg d^–1^ for adults by the World Health Organization (WHO) (Thomson, 2004). Therefore, to increase human Se intake, soil and foliar applications of Se-enriched fertilizers have been implemented in many areas worldwide (Ngigi et al., 2019), including Finland and New Zealand (Lyons et al., 2003).

Se uptake by plants is not only largely dependent on the total Se content of the external media; other factors such as pH, Eh, and Se species are also involved (Blaylock and James, 1994; Silva et al., 2019; Wang et al., 2020). In soil environments, Se is mainly present as selenate in oxidized and alkaline conditions (pe + pH > 15), whereas selenite is predominant in reduced and acidic to neutral states (7.5 < pe + pH < 15) (Elrashidi et al., 1987), such as in paddy soils (Diyabalanage et al., 2016). The absorption mechanisms of these compounds are considerably different: selenate is taken up by plants through sulfate transporters owing to the chemical similarity between selenate and sulfate (Terry et al., 2000; Sors et al., 2005), but the process of selenite uptake is still not fully understood. Previous studies have shown that the absorption of selenite could be affected by the presence of phosphate (P) in the media (Hopper and Parker, 1999). Zhang et al. (2014) reported that *OsPT2*, a phosphate transporter, is involved in selenite uptake, thereby indicating that a competitive relationship might exist between P and Se. Studies of wheat have also suggested that selenite absorption in plants was suppressed by increasing P supply (Liu et al., 2018) and was stimulated by P deficiency (Li et al., 2008). However, the contradictory results demonstrated that P supply elevated the Se concentration in alfalfa (Carter et al., 1972) and berseem (Singh and Malhotra, 1976). Given the inconsistency of previous findings, further studies on the absorption mechanisms of selenite as mediated by P in plants are still needed, and the process of Se translocation from roots to shoots should also be evaluated.

Studying the subcellular distribution and speciation of Se is fundamental to understanding the mechanisms underlying Se transport and metabolism in plants. In recent decades, numerous studies have investigated the subcellular distributions of various heavy metals (such as cadmium and lead) to reveal the tolerance and detoxification strategies of plants (Sha et al., 2019; Xiao et al., 2019; Yang et al., 2019). Other studies also focused on nutrient elements (Grillet et al., 2014; Srivastava et al., 2018) and rare-earth elements (Shen et al., 2014). Nonetheless, a few studies have reported about the subcellular Se distribution and accumulation in plants. Although Se speciation varies by plant species, it is commonly believed that organoselenium compounds in plants are superior to selenate and selenite regarding their safety and bioavailability to humans (Thiry et al., 2012); moreover, these compounds are also crucial in reducing the risk of cancer (Rayman, 2005). Hence, sufficient knowledge of Se speciation in plants is required when assessing the effects of Se biofortification in humans. Recently, Liu et al. (2018) adopted a sequential extraction method to determine the chemical forms of Se in wheat plants. However, these forms are considerably different from the actual Se species associated with Se metabolism compounds in plant tissues that can be extracted by enzymatic hydrolysis.

Rice (*Oryza sativa* L.) is a major cereal in Southeast and East Asia, and approximately 160 million hectares of land are dedicated to its production globally (Seck et al., 2012). Thus, rice can serve as an ideal candidate for human Se intake. The objectives of this hydroponic study were to: (1) ascertain the effect of additional P on Se uptake and translocation in rice seedlings under P-normal and P-deficient cultivation conditions; (2) explore the influence of distinct P regimes on the subcellular fractionation and speciation of stored Se in rice plants; and (3) provide theoretical guidance on fertilization strategies associated with Se biofortification of rice.

## Materials and Methods

### Plant Materials and Culture Conditions

Rice (*Oryza sativa* L. cv. Zhunliangyou 608) seeds were sterilized with 30% (v/v) H_2_O_2_ for 15 min, then thoroughly rinsed with deionized water and soaked in a saturated CaSO_4_ solution overnight. The prepared seeds were germinated on a floating pre-sterilized plastic sheet net moistened with distilled water at 25°C in darkness. Ten days after germination, the healthy and uniform seedlings were transferred to 2.5-L polyvinyl chloride (PVC) pots (four plants per pot) containing 1/2-strength Kimura solution. The composition of the nutrient solution was as follows: KNO_3_ 91, Ca(NO_3_)_2_⋅4H_2_O 183, MgSO_4_⋅7H_2_O 274, KH_2_PO_4_ 100, (NH_4_)_2_SO_4_ 183, MnSO_4_⋅H_2_O 1.0, H_3_BO_3_ 3.0, (NH_4_)_6_Mo_7_O_24_⋅4H_2_O 1.0, ZnSO_4_⋅7H_2_O 1.0, CuSO_4_⋅5H_2_O 0.2, and Fe(III)-EDTA 60 (μmol L^–1^). The solution pH was adjusted to 5.5 using diluted KOH and HCl and buffered with 2 mmol L^–1^ 2-(*N*-Morpholino) ethanesulfonic acid monohydrate (MES). The solution was renewed every 3 days. The rice plants were cultured in a greenhouse maintaining constant conditions throughout the entire experiment: 25 ± 4°C/20 ± 2°C (day/night), a 14 h d^–1^ photoperiod, a photon flux of 240–350 μmol m^–2^ s^–1^, and a relative humidity of 60–70%.

### Experimental Design

At 21 days after transplantation, the rice seedlings were divided into two groups. One group of seedlings was pre-cultured under P-normal conditions for 7 days and then supplied with additional P (1 mmol L^–1^, KH_2_PO_4_) and Se (50 μg L^–1^, Na_2_SeO_3_) to form four treatments: (1) –P–Se (without additional P and Se); (2) +P–Se (with additional P and without Se); (3) –P + Se (without additional P and with Se); and (4) + P + Se (with additional P and Se) in P-normal solution. The other group of seedlings was pre-cultured under P-deficient conditions (KH_2_PO_4_ was replaced by KCl) for 7 days and then treated with the same four treatments as above in P-deficient solution. The composition of other nutrients was consistent with that in the P-normal solution. Each treatment was replicated in three pots (two plants per pot). The seedlings were harvested after 3 days of treatments, then fixed with a pre-chilled desorption solution (1 mmol L^–1^ CaSO_4_, 2 mmol L^–1^ MES) for 15 min to remove P and Se in the root apoplast. After being thoroughly washed with deionized water, the separated shoots and roots of the plants were weighed. Next, they were frozen in liquid nitrogen, pulverized with a hammer, and stored at –80°C for elemental determination and Se speciation.

### Subcellular Fractions Separation

Approximately 0.4 g of fresh shoots and roots were homogenized with a mortar and pestle in 10 mL of a pre-cooled extraction buffer containing 1.0 mmol L^–1^ dithioerythritol, 250 mmol L^–1^ sucrose, and 50 mmol L^–1^ Tris-HCl (pH 7.5), according to the methods described by Wan et al. (2019) with some modifications. The homogenate was centrifuged at 300 *g* for 10 min, and the first residue was designated as the cell wall fraction (F1). The supernatant was then centrifuged at 20,000 *g* for 30 min, and the second precipitate and the final supernatant were designated as the organelle fraction (F2) and the soluble fraction (F3), respectively. All steps were performed at 4°C. The soluble fraction was diluted to 50 mL with 5% HNO_3_ (GR) prior to elemental determination.

### Element Analysis and Se Speciation Determination

A 0.4 g portion of fresh samples (i.e., shoots and roots) as well as the cell wall and organelle fractions were mineralized with 8 mL concentrated HNO_3_ (GR) overnight and then digested using a microwave oven (MARS5, CEM Corp, United States). The P concentration in the digested solution was determined using inductively coupled plasma-optical emission spectroscopy (ICP-OES; Perkin Elmer Optima 3300 DV), whereas the Se concentration was measured using a hydride generation-atomic fluorescence spectrometer (HG-AFS; Jitian Instruments Co., Beijing, China) after being pre-reduced with 6 mol L^–1^ HCl (GR) in a water bath at 95–99°C for 2 h. Blanks and a certified reference material (GSB-23, rice flour, Center for Standard Reference, PRC) were included in each batch of samples for quality control and assurance. The recovery of GSB-23 varied between 89 and 117%.

Selenium speciation in rice shoots and roots was extracted following the methods of Li et al. (2010) with some modifications. Briefly, 0.4 g of the frozen samples was extracted with 5 mL of 8 mg mL^–1^ protease XIV (Sigma Aldrich, United States) in 15 mL centrifugal tubes. The mixture was incubated in an oscillation box with horizontal shaking (125 rpm) at 37°C for 24 h. The hydrolyzate was centrifuged at 12,000 rpm for 15 min, and the resultant supernatant was filtered through a 0.22 μm cellulose nitrate filter (Millipore, Billerica, MA, United States). Subsequently, the filtrate was stored at –80°C for Se speciation analysis, which was conducted using high performance liquid chromatography-ultraviolet treatment-hydride generation-atomic fluorescence spectrometry (HPLC-UV-HG-AFS; SA-50, Jitian Instruments Co., Beijing, China).

The separation of Se species was performed using an anion exchange column (4.1 mm × 250 mm × 10 μm; PRP-X100; Hamilton, Switzerland) equipped with a guard column (2.3 mm × 25.0 mm × 10–20 μm; PRP-X100; Hamilton, Switzerland), in which the 100 μL samples were eluted by the mobile phase (pH 6.00, adjusted by 10% HCOOH) containing 40 mmol L^–1^ (NH_4_)_2_HPO_4_ at a flow rate of 1.0 mL min^–1^. To measure the amount of a certain Se species, the HPLC was in conjunction with a UV-HG-AFS. A UV lamp (78 W) was used to digest Se species on-line. The generation of hydrogen selenide in the HG parts was oxidized and reduced by 0.2% KI (m/v) and 2.0% KBH_4_ (m/v), both dissolved in 0.35% KOH (m/v) and carried by 10% HCl (v/v; GR). The operating conditions of the HG-AFS were as follows: 80 mA of lamp current, 285 V of negative high voltage, a 400 mL min^–1^ carrier gas rate, and a 600 mL min^–1^ shield gas rate. Five standard selenocompounds, i.e., selenocystine (SeCys_2_), Se-methyl-selenocysteine (MeSeCys), selenite [Se(IV)], selenomethionine (SeMet), and selenate [Se(VI)], purchased from the National Research Center for Certified Reference Materials, Beijing, China, were identified according to their retention times. Simultaneously, the identified Se species in the samples were quantified based on the peak areas of the calibration curves using a HPLC workstation.

### Data Analysis

Total elemental concentration (*T*_*Shoot*_, *T*_*Root*_ and *T*), elemental uptake by roots, and the transfer factor (*TF*) of P and Se were calculated using Equations (1–5):

(1) $$T_{S\,h\,o\,o\,t-P/S\,e}=C_{S\,h\,o\,o\,t-P/S\,e}\times B\,i\,o\,m\,a\,s\,s_{S\,h\,o\,o\,t}$$

(2) $$T_{R\,o\,o\,t-P/S\,e}=C_{R\,o\,o\,t-P/S\,e}\times B\,i\,o\,m\,a\,s\,s_{R\,o\,o\,t}$$

(3) $$T_{P/S\,e}=T_{S\,h\,o\,o\,t-P/S\,e}+T_{R\,o\,o\,t-P/S\,e}$$

(4) $$P/S\,e\,u\,p\,t\,a\,k\,e\,r\,a\,t\,e=T_{P/S\,e}/B\,i\,o\,m\,a\,s\,s_{R\,o\,o\,t}$$

(5) $$T\,F_{P/S\,e}=C_{S\,h\,o\,o\,t-P/S\,e}/C_{R\,o\,o\,t-P/S\,e}$$

where *C*_*Shoot–P/Se*_ and *C*_*Root*−*P*/*Se*_ represent the P or Se concentration in rice shoots and roots, respectively.

The proportion of Se subcellular distribution in rice tissues was calculated using Equations (6–7):

(6) $$Shoot_{F\,1/F\,2/F\,3}-Se\%=C_{S\,h\,o\,o\,t\,F\,1/F\,2/F\,3-S\,e}/\left(C_{S\,h\,o\,o\,t\,F\,1-S\,e}+C_{S\,h\,o\,o\,t\,F\,2-S\,e}+C_{S\,h\,o\,o\,t\,F\,3-S\,e}\right)$$

(7) $$Root_{F\,1/F\,2/F\,3}-Se\%=C_{R\,o\,o\,t\,F\,1/F\,2/F\,3-S\,e}/\left(C_{R\,o\,o\,t\,F\,1-S\,e}+C_{R\,o\,o\,t\,F\,2-S\,e}+C_{R\,o\,o\,t\,F\,3-S\,e}\right)$$

where*C*_*ShootF*1/*F*2/*F*3−*Se*_ and *C*_*RootF1/F2/F3–Se*_ represent the Se concentration in the cell wall, the organelle, and soluble fraction of the shoots and roots, respectively.

The proportion of each Se species (*Pro*_*i*_) in rice was calculated using Equation (8):

(8) $$P\,r\,o_{i}=C_{i}/\sum C_{i}\times 100\%$$

where *C*_*i*_ and ∑*C*_*i*_ represent the concentration of a certain Se species and the sum of the concentrations of the five Se species in the rice tissues, respectively.

One-way analysis of variance (ANOVA) was performed using SPSS 19.0 to determine the significance of the treatment effects. All data were calculated on a fresh weight (FW) basis, and the means were compared using Duncan’s multiple range test at the 5% level.

## Results

### Biomass, P Uptake, and Translocation Within Rice Seedlings

In general, the biomass of rice shoots slightly decreased under P-deficient cultivation, but a significant increase (*P* < 0.05) was observed in the roots compared to the biomass of the corresponding treatments under P-normal cultivation (Table 1). Moreover, neither the supply of additional P nor Se had pronounced effects (*P* > 0.05) on the fresh mass of shoots and roots under both P-deficient and P-normal conditions.

**TABLE 1:** Uptake, accumulation, and translocation of P in rice seedlings under different treatments.

	Treatment	Biomass (g pot^–1^ FW)		P concentration (mg g^–1^ FW)		P uptake rate (mg g^–1^ root FW)	Transfer factor (TF)
		Shoots	Roots	Shoots	Roots		
P-deficient	−P−Se	8.20 ± 0.31^*bc*^	4.45 ± 0.07^*a*^	0.41 ± 0.01^*d*^	0.25 ± 0.02^*e*^	1.00 ± 0.05^*d*^	1.63 ± 0.13^*ab*^
	+P−Se	9.28 ± 0.29^*abc*^	4.42 ± 0.11^*a*^	1.46 ± 0.10^*a*^	0.98 ± 0.01^*cd*^	4.04 ± 0.17^*bc*^	1.49 ± 0.10^*ab*^
	−P + Se	7.38 ± 0.95^*c*^	3.94 ± 0.24^*abc*^	0.45 ± 0.02^*d*^	0.26 ± 0.01^*e*^	1.10 ± 0.09^*d*^	1.75 ± 0.11^*a*^
	+P+Se	8.85 ± 0.83^*abc*^	4.34 ± 0.36^*ab*^	1.31 ± 0.05^*ab*^	0.95 ± 0.00^*d*^	3.63 ± 0.10^*c*^	1.39 ± 0.05^*b*^
P−normal	−P−Se	9.37 ± 0.63^*abc*^	3.21 ± 0.21^*c*^	1.05 ± 0.00^*c*^	1.18 ± 0.03^*ab*^	4.24 ± 0.03^*ab*^	0.89 ± 0.02^*c*^
	+P−Se	9.47 ± 0.37^*ab*^	3.62 ± 0.12^*bc*^	1.27 ± 0.05^*b*^	1.18 ± 0.03^*ab*^	4.51 ± 0.22^*ab*^	1.07 ± 0.03^*c*^
	−P + Se	10.60 ± 0.46^*a*^	3.75 ± 0.29^*abc*^	1.16 ± 0.06^*bc*^	1.10 ± 0.09^*bc*^	4.39 ± 0.09^*ab*^	1.08 ± 0.15^*c*^
	+P + Se	9.52 ± 0.69^*ab*^	3.41 ± 0.23^*c*^	1.21 ± 0.06^*bc*^	1.25 ± 0.08^*a*^	4.63 ± 0.27^*a*^	0.97 ± 0.06^*c*^

*Data are presented as means ± standard errors (SEs; *n* = 3). Different letters in the same column indicate statistically significant differences among the treatments at *P* < 0.05 according to Duncan’s test.*

The P concentration and uptake rate of roots exposed to P deficiency were much lower than those of roots exposed to P-normal cultivation, and these factors were elevated by 2.9 and 3.0 times, respectively, when only additional P was supplied to the P-deficient solution. However, this increase was not observed under the P-normal condition. P deficiency sharply enhanced (*P* < 0.05) the P transfer factor by 39–83% in rice seedlings, whereas additional P supply had no significant effect (*P* > 0.05) on this factor under P-deficient and P-normal conditions. Furthermore, P deficiency generally reduced the shoot P concentration compared to that of the corresponding treatments under P-normal conditions, although the +P–Se treatment was an exception, with a 15% increase. The additional P supply increased the shoot P concentration in exposures to both culture conditions, regardless of Se addition. However, no obvious changes (*P* > 0.05) were caused by Se addition to each nutrient solution regarding P uptake and translocation in rice plants.

### Effect of P on Se Uptake and Translocation in Rice Seedlings

**FIGURE 1:**
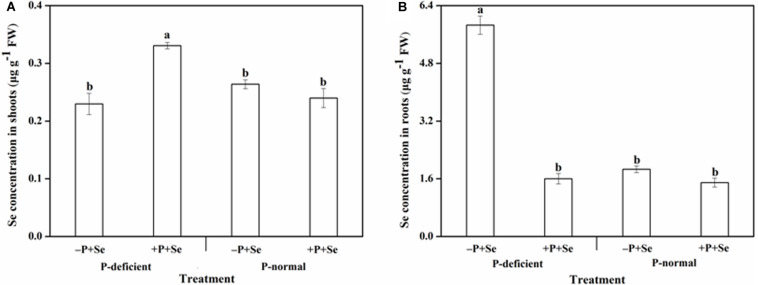
Se concentration in **(A)** shoots and **(B)** roots of rice seedlings under different treatments. Data are presented as means ± SEs (*n* = 3). Different letters indicate statistically significant differences among the treatments at *P* < 0.05 according to Duncan’s test.

The Se concentration of rice tissues was clearly distinct among the different treatments (Figure 1). The highest Se concentration in the shoots was 0.33 μg g^–1^ under the P-deficient + P + Se treatment, while for the roots, the Se concentration was invariably more than 1.40 μg g^–1^ and reached a maximum value of 5.86 μg g^–1^ under the P-deficient –P + Se treatment.

The Se concentration in rice shoots showed similar responses when exposed to the P-deficient and P-normal solutions without additional P (Figure 1A). Similarly, no obvious effects (*P* > 0.05) related to P addition were found on the Se concentration under P-normal condition. Nevertheless, the additional P supply greatly increased (*P* < 0.05) the shoot Se concentration by 44% relative to the –P + Se treatment under P deficiency. On the other hand, the Se concentration of roots exposed to the P-deficient –P + Se treatment was significantly increased (*P* < 0.05) by 2.1 times compared with that of the P-normal condition (Figure 1B). Moreover, the additional P supply slightly decreased the root Se concentration by 20% compared to that of the –P + Se treatment under P-normal condition; however, this effect was more profound under P deficiency, with decreases of up to 73%.

**FIGURE 2:**
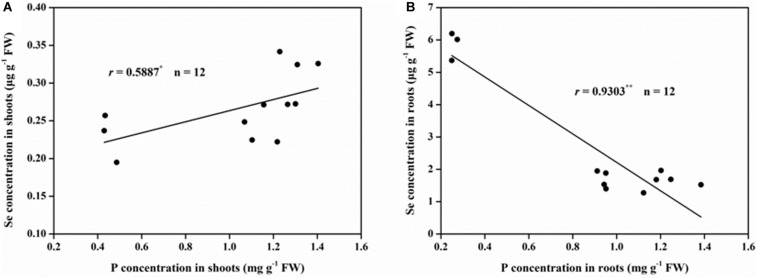
Correlations between Se and P concentration in rice **(A)** shoots and **(B)** roots. ^∗^Correlation is significant at *P* < 0.05; ^∗∗^Correlation is significant at *P* < 0.01.

There was a positive correlation between the shoot Se and P concentration, which can be described by a linear curve (*r* = 0.5887, *P* < 0.05; Figure 2A). However, a very significantly negative and linear relationship (*r* = 0.9303, *P* < 0.01; Figure 2B) was found between the Se and P concentration observed in the roots.

**FIGURE 3:**
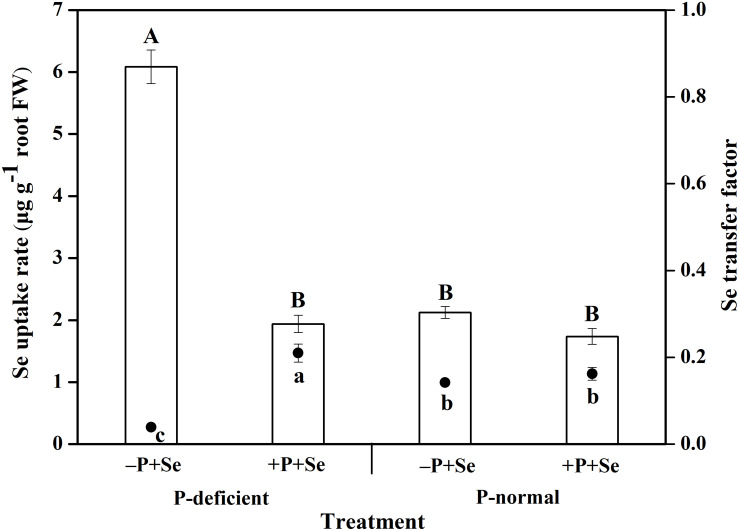
The uptake and translocation of Se in rice seedlings under different treatments. The histogram and scatter diagram depict the Se uptake rate and transfer factor in rice, respectively. Data are presented as means ± SEs (*n* = 3). The different capital and lowercase letters indicate statistically significant differences in Se uptake and translocation, respectively, among the treatments at *P* < 0.05 according to Duncan’s test.

P deficiency and the additional P supply had significant effects on Se uptake and translocation within rice seedlings (Figure 3). The Se uptake by rice cultured with the –P + Se treatment under P deficiency was 1.4 times higher than that under the P-normal condition. Furthermore, relative to Se treatment alone, the co-supply of additional P significantly inhibited (*P* < 0.05) the Se uptake by 64% under P-deficient condition, but this inhibition was only 17% under P-normal condition. On the other hand, the Se transfer factor from roots to shoots under the P-deficient –P + Se treatment was obviously reduced (*P* < 0.05) by 72% relative to that under the P-normal condition. In response to P addition to the P-deficient solution, Se translocation was stimulated by 4.4 times. However, only a minor increase of 14% was found in the Se transfer factor after P was added to the P-normal solution.

### Effect of P on Se Subcellular Distribution in Rice

As shown in Table 2, Se concentration in the cell wall (F1) of the shoots was far higher than that in the organelle (F2) and soluble fraction (F3), and was more sensitive to P deficiency or additional P treatment. With respect to the –P + Se treatment, P deficiency significantly decreased (*P* < 0.05) the Se level in the shoot cell wall by 39% compared to that under the P-normal condition. Furthermore, additional P remarkably decreased the Se concentration in the cell wall by 33% relative to the Se-only treatment under P-normal condition, but significantly increased it by 1.2 times under P-deficient condition (*P* < 0.05). Contrarily, neither P-deficient cultivation nor additional P treatment influenced the Se concentration in the organelle and soluble fraction of shoots to any great extent.

**TABLE 2:** Subcellular distribution of Se in rice seedlings under different treatments.

		Treatment	Se concentration (μg g^–1^ FW)		
			F1	F2	F3
Shoots	P-deficient	–P + Se	0.11 ± 0.02^*b*^	0.01 ± 0.00^*a*^	0.02 ± 0.01^*a*^
		+P+Se	0.24 ± 0.02^*a*^	0.03 ± 0.01^*a*^	0.03 ± 0.01^*a*^
	P-normal	–P + Se	0.18 ± 0.01^*a*^	0.03 ± 0.00^*a*^	0.01 ± 0.00^*a*^
		+P+Se	0.12 ± 0.00^*b*^	0.02 ± 0.00^*a*^	0.02 ± 0.00^*a*^
Roots	P-deficient	–P + Se	4.21 ± 0.11^*A*^	0.45 ± 0.06^*A*^	0.65 ± 0.03^*A*^
		+P+Se	0.97 ± 0.10^*BC*^	0.10 ± 0.02^*B*^	0.41 ± 0.04^*B*^
	P-normal	–P + Se	1.19 ± 0.06^*B*^	0.14 ± 0.02^*B*^	0.59 ± 0.04^*A*^
		+P+Se	0.69 ± 0.17^*C*^	0.11 ± 0.05^*B*^	0.36 ± 0.07^*B*^

*F1, F2, and F3 represent the cell wall, organelle, and soluble fraction, respectively. Data are presented as means ± SEs (*n* = 3). The different lowercase or capital letters in the same column indicate statistically significant differences among the treatments at *P* < 0.05 according to Duncan’s test.*

The Se concentration in different subcellular fractions of the roots varied greatly among the treatments, but the overall pattern can be ranked as follows: cell wall > soluble fraction > organelle (Table 2). Compared to the effects observed under P-normal condition, P deficiency combined with –P + Se treatment significantly increased (*P* < 0.05) the Se concentration in the root cell wall and organelle by 2.5 and 2.2 times, respectively, but it had no obvious effect on that of the soluble fraction. However, the co-supply of additional P induced a general decline in the root subcellular Se concentration (77% for F1, 78% for F2, and 37% for F3), compared to the Se-only supply under P-deficient condition; the decreases in root Se concentration under P-normal condition were 42% for the cell wall and 39% for the soluble fraction, with only a slight change for the organelle.

**FIGURE 4:**
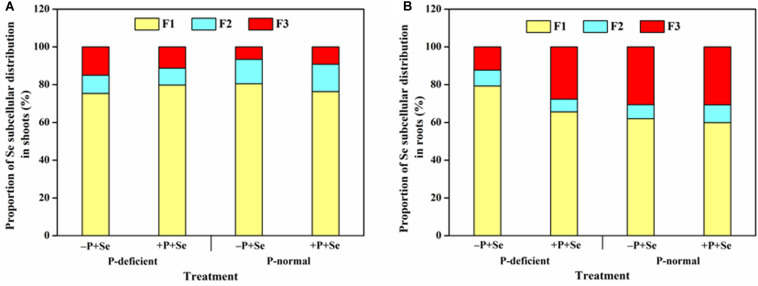
Proportion of Se in different subcellular fractions of **(A)** shoots and **(B)** roots under different treatments. F1, F2, and F3 represent the cell wall, organelle, and soluble fraction, respectively. Data are presented as the means of three independent replicates.

With respect to all the treatments, the absorbed Se was mainly deposited in the cell wall of rice shoots (>75%) and roots (>60%). P deficiency decreased the Se percentage in the cell wall and organelle in the shoots but increased it by 1.3 times in the soluble fraction, compared with that of the –P + Se treatment under P-normal condition (Figure 4A). Relative to the Se-only treatment, adding P to the P-normal solution reduced Se sequestration by the cell wall and increased the distribution ratio of the soluble fraction in the shoots, while the opposite was true under P-deficient condition. On the other hand, P deficiency enhanced Se storage in the root cell wall by 28%, whereas it significantly decreased (*P* < 0.05) the Se percentage in the soluble fraction by 60% relative to that observed in the –P + Se treatment under P-normal condition (Figure 4B). Furthermore, compared with the Se-only treatment, the co-supply of additional P remarkably increased the Se percentage by 1.3 times in the soluble fraction of roots exposed to the P-deficient solution, while only minor changes were induced by additional P with respect to the Se subcellular distribution under P-normal condition.

### Effect of P on Se Species in Rice

**FIGURE 5:**
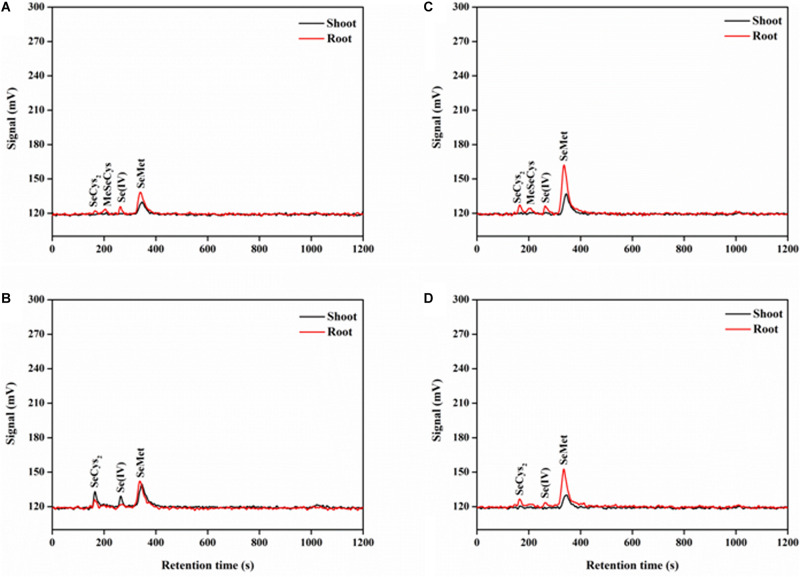
Examples of chromatograms of Se species in protease XIV extracts of rice tissues, as determined by anion exchange HPLC-UV-HG-AFS. Se species of rice seedlings in **(A)** –P + Se and **(B)** + P + Se treatments under P-deficient condition. Se species of rice seedlings in **(C)** –P + Se and **(D)** + P + Se treatments under P-normal condition. SeCys_2_, selenocystine; MeSeCys, Se-methyl-selenocysteine; Se(IV), selenite; SeMet, selenomethionine.

The separation of the five standard selenocompounds was highly accurate through HPLC-UV-HG-AFS under the current working conditions (Supplementary Figure S1). The retention time of SeCys_2_, MeSeCys, Se(IV), SeMet, and Se(VI) was approximately 169, 206, 272, 341, and 1,028 s, respectively. After the protease XIV extraction, all Se species detected in rice tissues were classified within the five standard selenocompounds (Figure 5). Generally, only SeMet was detected in rice shoots, except for the P-deficient + P + Se treatment. On the contrary, four Se species [i.e., SeCys_2_, MeSeCys, Se(IV), and SeMet] were detected in the roots. However, Se(VI) was not detected in any of the selenite-treated samples.

**FIGURE 6:**
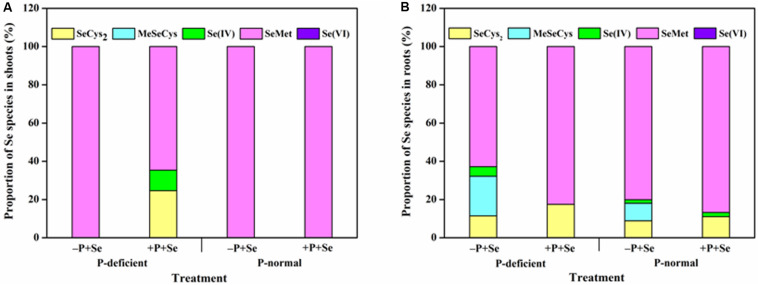
Proportion of Se species in **(A)** shoots and **(B)** roots of rice seedlings under different treatments. SeCys_2_, selenocystine; MeSeCys, Se-methyl-selenocysteine; Se(IV), selenite; SeMet, selenomethionine; Se(VI), selenate. Data are presented as the means of three independent replicates.

The most abundant Se species was SeMet, which accounted for more than 60% of the total Se detected in rice tissues, regardless of treatment (Figure 6). Only SeMet was detected in rice shoots in most cases, while SeCys_2_ and Se(IV) were also detected under the P-deficient + P + Se treatment (Figure 6A). For roots treated with only selenite, P deficiency significantly reduced (*P* < 0.05) the proportion of SeMet by 22% but increased that of SeCys_2_, MeSeCys, and Se(IV) by 29, 125, and 170%, respectively, as compared to the proportions under P-normal condition (Figure 6B). Under P-normal condition, the proportion of SeCys_2_ and SeMet was elevated by 24 and 8%, respectively, while that of MeSeCys was reduced by the co-supply of additional P. Moreover, compared to the Se-only treatment, the proportion of Se(IV) in the roots was also reduced when additional P was added to the P-deficient solution, thereby leading to increased proportions of organic Se (53% for SeCys_2_ and 31% for SeMet). However, MeSeCys was not detected under the P-deficient + P + Se treatment.

## Discussion

Although Se is not an essential element for plants, it is beneficial for their growth if applied at a proper dose (Hartikainen, 2005; Shalaby et al., 2017). However, the results of this study showed that Se supply had no obvious effect on the rice biomass (Table 1), nor did the additional P, even though P is critical in improving crop yields in agricultural practices. The short exposure time (3 days) or the low dosages of Se and P applied in this experiment may help explain this phenomenon. Nevertheless, P-deficient cultivation induced a remarkable increase in the fresh weight of rice roots but a slight decrease in the shoots, compared to the weight of the corresponding treatments under P-normal condition. These findings are consistent with other observations concerning *Arabidopsis* (Gruber et al., 2013). It has been revealed that with the involvement of phytohormone synthesis, P starvation inhibits the growth of primary roots, while stimulates the formation and elongation of lateral roots and increases the root:shoot biomass ratio in plants, thus improving their ability to extract P from their surroundings (Nacry et al., 2005; Hermans et al., 2006).

Earlier studies reported that Se accumulation in plants was affected by the status of P nutrition in the external media (Carter et al., 1972; Mora et al., 2008; Liu et al., 2018). As expected, the results of this study showed that P deficiency combined with –P + Se treatment significantly increased (*P* < 0.05) the Se concentration of rice roots; however, the co-supply of additional P with Se decreased the Se concentration under two cultivation cultures (Figure 1B). Similar results were achieved by Liu et al. (2004, 2018) and Li et al. (2019), who found that the root Se concentration gradually declined with increasing P concentrations in the nutrient solution. Meanwhile, in both P-deficient and P-normal conditions, the Se uptake was inhibited when additional P was applied to the cultures (Figure 3). It was reported that *OsPT2*, a phosphate transporter, is involved in the active uptake of selenite in rice plants (Zhang et al., 2014). Consequently, antagonism might exist between the absorption of P and Se from the media to the roots, as suggested by the negative correlation between the root P and Se concentration (Figure 2B). Nevertheless, the inverse tendency was achieved in field or pot experiments. For instance, the Se uptake of barley, potato, and wheat grown in high P-availability soil was much greater than those in low P-availability soil (Altansuvd et al., 2014), and P addition to the soil enhanced the Se uptake of alfalfa grown in six soil types (Carter et al., 1972). Therefore, the specific effect of P on Se uptake by plants depends both on the P dosage as well as the environmental conditions.

The translocation of Se from roots to shoots is highly dependent on the form in which Se is supplied. Zayed et al. (1998) reported that the Se shoot/root ratio ranged from 1.4 to 17.2 for plants supplied with selenate, while this ratio was less than 0.5 for those treated with selenite. Similarly, the Se transfer factor in rice treated by selenite was no more than 0.21 in our study (Figure 3), suggesting that most of the Se accumulated in roots and was seldom transported to the upper parts of the plants. Additionally, Se translocation in the rice treated with only selenite was reduced by P deficiency (Figure 3), whereas the opposite effect was found for P translocation (Table 1). This phenomenon might be attributable to the competition between P and Se during plant transport, and as P is an important macronutrient for plants, it might be transported preferentially, especially under P-deficient condition. Previous studies have shown that *OsPT6* likely mediates P translocation throughout the plants and is strongly up-regulated under P starvation (Luan et al., 2017; Srivastava et al., 2018). The transcript abundance of *OsPT6* was notably elevated in the roots of an *ltn1* mutant, which exhibited much higher rates of Se uptake than wide-type plants (Zhang et al., 2014). Therefore, it is rational to speculate that *OsPT6* is involved in both P and Se translocation in rice. Furthermore, a 4.4-fold increase in the Se transfer factor in the present study was caused by the co-supply of additional P compared to that under the P-deficient Se-only treatment (Figure 3). Evidence has shown that selenite is reduced to selenide non-enzymatically by glutathione (GSH) in plants (Terry et al., 2000; Sors et al., 2005) and is then transformed into organic forms and transported to the shoots. Given that the synthesis of GSH requires ATP (Lu, 2013), we speculate that additional P supply may be conducive to GSH synthesis and Se transformation, thereby enhancing Se translocation within rice seedlings. Overall, Se translocation is postulated to be a carrier-mediated and ATP-requiring process, and future studies are needed to further investigate the translocation mechanisms of Se.

Accompanying with the elevated Se transfer factor (Figure 3), the shoot Se concentration was stimulated by the co-supply of additional P under P deficiency (Figure 1A). This finding is partly supported by Li et al. (2019), who reported that the Se concentration in shoots was elevated when rice was grown in a medium with increased P levels. Moreover, Se accumulation in shoots can also be affected by its subcellular distribution within rice. In the present study, more than 75 and 60% of Se was distributed in the cell wall of shoots and roots, respectively. This result is in accordance with the previous studies that demonstrated Se was primarily deposited in the cell wall fraction of rice (Ding et al., 2015; Liu et al., 2018; Dai et al., 2019).

Plants have developed a known series of defense mechanisms to against the stress caused by toxic elements, such as binding said elements to cell walls or sequestering them in vacuoles (Baker, 1987). As the first cellular barrier, the cell wall restrains the movement of ions across the cell membrane by binding and retention (Haynes, 1980; Gallego et al., 2012). Moreover, the cell vacuole serves as a major organelle that can fix and accumulate ions by incorporating them with its internal organic acids and sulfuric peptides, and the vacuole is central to inhibiting the entrance of ions into the xylem and the translocation to shoots (Fu et al., 2011; Nocito et al., 2011). Selenium can act as an antioxidant that directly stimulates the antioxidative capacity of organisms (D’ Amato et al., 2019) or by improving the activities of antioxidant enzymes like superoxide dismutase (SOD) (Hasanuzzaman et al., 2010). However, the antioxidant enzymes were observed as being inhibited by a high Se concentration (Assunção et al., 2015); thus, Se can be considered as both a nutrient and toxin to mammals and plants (Kaur et al., 2014). Additionally, as a narrow boundary exists between beneficial and toxic concentrations of Se, more attention should be paid to the Se subcellular distribution. In the rice roots, P deficiency combined with –P + Se treatment significantly strengthened Se binding in the cell wall but reduced the Se distribution in the soluble fraction, compared to that under the P-normal condition. In contrast, the co-supply of additional P under P deficiency increased the Se proportion in the soluble fraction despite the general decline of Se concentration in all root fractions (Figure 4B). These results suggest that P supply could facilitate Se passing through the cell membrane and being transported to the aerial parts of rice grown in P-deficient solutions, which is in accordance with the findings of Se translocation in this study (Figure 3). Contrarily, in rice shoots, P supply enhanced Se storage in the cell wall but reduced its relative distribution in the soluble fraction (Figure 4A), which was also observed by Liu et al. (2018).

After being absorbed by plant roots, Se is converted to organic forms through sulfur (S) metabolic pathway, with selenocysteine (SeCys), SeMet, and MeSeCys being the main seleno-amino acids (Whanger, 2002; Sors et al., 2005; Rayman et al., 2008). SeCys is synthesized via the donation of a carbon skeleton from *O*-acetylserine to selenide through the action of the Cys synthase (Ng and Anderson, 1978); then, it can be further methylated to form either SeMet or MeSeCys (Lyi et al., 2005; White, 2016). In this study, the most abundant Se species detected in rice tissues was SeMet (Figure 6). Considering the high bioavailability and excellent cancer-preventing effects of SeMet (Rayman, 2005; Rayman et al., 2008), rice is an ideal medium to enhance Se dietary intake, thus strengthening human physique and cancer resistance. Our results are consistent with the findings of Dai et al. (2019) concerning rice leaves and young spikes. However, various results have been found for other plants, or even distinct parts of the same plant. For example, selenomethionine-Se-oxide (SeOMet) appeared to be the most abundant form of Se in selenite-treated Indian mustard (Kahakachchi et al., 2004); *Se*-methylselenocysteine (SeMeSeCys) was the major Se species in Se-enriched onion, leek, and broccoli (Beilstein et al., 1991). Furthermore, Yu et al. (2019) reported that SeMet was the dominant Se species in roots, while SeMeCys in shoots of pak choi treated with selenite.

Generally, only SeMet was detected in rice shoots, while other Se species were not observed in the present study (Figure 6A); this result was likely owed to the low total Se concentration of shoots (Figure 1A). An exception existed for the +P + Se treatment under P-deficient condition, in which the higher total Se concentration induced by the additional P supply might be the main reason why SeCys_2_ and Se(IV) were also detected. Additionally, the presence of Se(IV) suggested that selenite was not entirely transformed into other species, since its conversion is not irreversible in rice seedlings. Unlike the shoots, considerable amounts of various organic selenocompounds were detected in the roots, including SeCys_2_, MeSeCys, and SeMet (Figure 6B); similar results were achieved for wheat by Huang et al. (2017) and Hu et al. (2018) and for pak choi by Yu et al. (2019). The formation of these seleno-amino acids is known to occur in the chloroplasts of plants (Ng and Anderson, 1979; Brühl et al., 1996; Terry et al., 2000). In certain circumstances, chloroplasts can be induced in roots via genetic control (Oliveira, 1982); accordingly, inorganic Se was most likely transformed in root chloroplasts. In the present study, the co-supply of additional P induced an increase in the total proportion of SeCys_2_ and SeMet in roots under P-deficient condition. Given the greater translocation of organoselenium than for inorganic forms (Zayed et al., 1998), the increased P supply likely stimulated Se translocation from roots to shoots when exposed to P deficiency, as is indicated by our findings presented in Figure 3. It is interesting to note that P deficiency combined with –P + Se treatment decreased the proportion of SeMet but elevated that of MeSeCys in roots, compared to those under P-normal condition. However, the opposite tendencies were achieved for the +P + Se treatment under both cultivation conditions (Figure 6B). MeSeCys is synthesized via the methylation of SeCys, while SeMet is produced from SeCys via SeCystathionine and SeHomoCys, and a series of enzymes (i.e., Cystathionine-γ-synthase, Cystathionine-β-lyase, and Met synthase) are involved in these successive reactions (Terry et al., 2000). In light of P plays an important role in protein synthesis and affects the activities of various enzymes, the SeMet biosynthesis might be inhibited by the transformation of the precursor SeCys into MeSeCys within the roots when exposed to P deficient cultivation. However, further verification of this possibility is still needed.

Applying Se-enriched fertilizers to improve the Se content in the edible parts of crops is an effective long-term strategy in agricultural practices (Broadley et al., 2010; Ramkissoon et al., 2019). In the current study, both the Se accumulation and speciation in rice plants were significantly affected by distinct P regimes, which can give us a hint how to balance the P and Se in agricultural fields during fertilization applications. Based on the obtained results, we suggest the Se-biofortified rhizomatous plants seem to be suitable for planting in low P-availability soil. On the other hand, the supply of P fertilization in P-deficient soil is more feasible for cultivating cereal crops (such as wheat and rice) to enhance the Se concentration in their grains, especially SeMet, and thus meeting the daily dietary requirements. However, these strategies may be inappropriate for normal-P or high-P regions because of the poor efficiency of Se biofortification. It should also be noted that excessive P fertilization may inhibit Se accumulation in grains (Liu et al., 2018), as well as lead to severe environmental problems in intensive agricultural areas, like eutrophication and heavy metal pollution, which have received considerable attention in China (Le et al., 2010; Parameswari et al., 2014). Therefore, we view the specific P and Se fertilizer strategies are closely correlated to local soil characteristics and plant species.

## Conclusion

The present study verified that P nutritional conditions had significant effects on Se absorption and translocation in rice seedlings. The co-supply of additional P decreased the Se concentration in roots but increased it in shoots by improving Se translocation when cultured under P-deficient condition. Such effects could be ascribed to the enhancement of the Se distribution in cellular soluble fraction and the increase of the proportions of SeCys_2_ and SeMet in rice roots. However, no obvious effects of additional P supply were found in the case of P-normal cultivation. These results provide critical information to support the rational application of P fertilization and the effective agricultural production of Se-enriched rice.

## Data Availability Statement

All datasets generated for this study are included in the article/Supplementary Material.

## Author Contributions

YqW, KW, QW, and HL conceived and designed the experiments. YqW and KW performed the experiments. YqW analyzed the data and wrote the manuscript. KW, YnW, ZZ, YY, and HL reviewed and edited the manuscript. All the authors read and agreed with the final manuscript.

## Conflict of Interest

The authors declare that the research was conducted in the absence of any commercial or financial relationships that could be construed as a potential conflict of interest.
