# Supplementary material for: Selenite Uptake and Transformation in Rice Seedlings (Oryza sativa L.): Response to Phosphorus Nutrient Status
Source: Front Plant Sci. 2020 Jun 23;11:874. doi: 10.3389/fpls.2020.00874 (PMC7324753; doi:10.3389/fpls.2020.00874)
Supplement: Supplementary file 1 [file Image_1.pdf]

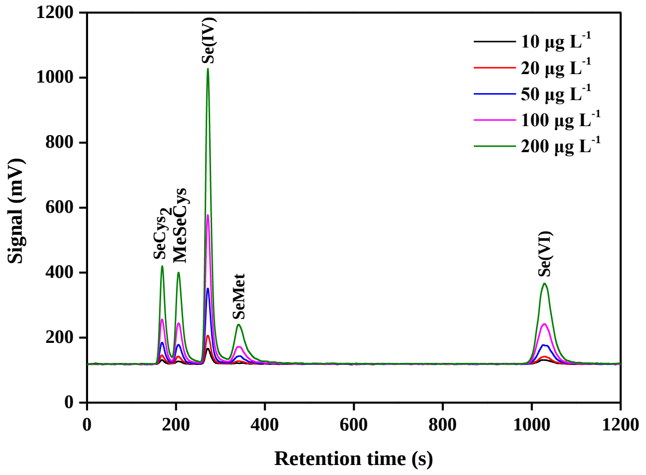


**Figure S1 |** Chromatogram of five standard selenocompounds determined via HPLC-UV-HG-AFS. $SeCys_2$, selenocystine; MeSeCys, Se-methyl-selenocysteine; Se(IV), selenite; SeMet, selenomethionine; Se(VI), selenate.
